# Polyphenolic Extract of *Euphorbia supina* Attenuates Manganese-Induced Neurotoxicity by Enhancing Antioxidant Activity through Regulation of ER Stress and ER Stress-Mediated Apoptosis

**DOI:** 10.3390/ijms18020300

**Published:** 2017-01-30

**Authors:** Entaz Bahar, Geum-Hwa Lee, Kashi Raj Bhattarai, Hwa-Young Lee, Min-Kyung Choi, Harun-Or Rashid, Ji-Ye Kim, Han-Jung Chae, Hyonok Yoon

**Affiliations:** 1 College of Pharmacy, Research Institute of Pharmaceutical Sciences, Gyeongsang National University, Jinju 52828, Gyeongnam, Korea; entaz_bahar@yahoo.com; 2 Department of Pharmacology, Medical School, Chonbuk National University, Jeonju 54896, Jeonbuk, Korea; heloin@jbnu.ac.kr (G.-H.L.); meekasik@jbnu.ac.kr (K.R.B.); youngat84@gmail.com (H.-Y.L.); mkelf78@nate.com (M.-K.C.); rashid@jbnu.ac.kr (H.-O.R.); 3 Department of Pathology, Severance Hospital and Yonsei University College of Medicine, Seoul 03722, Korea; alucion@gmail.com

**Keywords:** manganese, *Euphorbia supina*, neurotoxicity, antioxidant, neuroprotection

## Abstract

Manganese (Mn) is an important trace element present in human body, which acts as an enzyme co-factor or activator in various metabolic reactions. While essential in trace amounts, excess levels of Mn in human brain can produce neurotoxicity, including idiopathic Parkinson’s disease (PD)-like extrapyramidal manganism symptoms. This study aimed to investigate the protective role of polyphenolic extract of *Euphorbia supina* (PPEES) on Mn-induced neurotoxicity and the underlying mechanism in human neuroblastoma SKNMC cells and Sprague-Dawley (SD) male rat brain. PPEES possessed significant amount of total phenolic and flavonoid contents. PPEES also showed significant antioxidant activity in 1,1-diphenyl-2-picrylhydrazyl (DPPH) radical scavenging and reducing power capacity (RPC) assays. Our results showed that Mn treatment significantly reduced cell viability and increased lactate dehydrogenase (LDH) level, which was attenuated by PPEES pretreatment at 100 and 200 µg/mL. Additionally, PPEES pretreatment markedly attenuated Mn-induced antioxidant status alteration by resolving the ROS, MDA and GSH levels and SOD and CAT activities. PPEES pretreatment also significantly attenuated Mn-induced mitochondrial membrane potential (ΔΨm) and apoptosis. Meanwhile, PPEES pretreatment significantly reversed the Mn-induced alteration in the GRP78, GADD34, XBP-1, CHOP, Bcl-2, Bax and caspase-3 activities. Furthermore, administration of PPEES (100 and 200 mg/kg) to Mn exposed rats showed improvement of histopathological alteration in comparison to Mn-treated rats. Moreover, administration of PPEES to Mn exposed rats showed significant reduction of 8-OHdG and Bax immunoreactivity. The results suggest that PPEES treatment reduces Mn-induced oxidative stress and neuronal cell loss in SKNMC cells and in the rat brain. Therefore, PPEES may be considered as potential treat-ment in Mn-intoxicated patients.

## 1. Introduction

Manganese (Mn) is a vital trace element for normal function and development of human body [1]. Mn binds to and/or regulates several important body enzymes such as Mn-superoxide dismutase (Mn-SOD) and pyruvate carboxylase in the growth and development of central nervous system (CNS) [2]. In micronutrient studies, Mn deficiency has been found in parenteral nutrition patients [3,4]. Exposure to excess levels of Mn produces cognitive, psychiatric, and motor abnormalities [3]. It has been reported that overexposure to the Mn could produce neurodegenerative damage, resulting in development of manganism symptoms such as cognitive, psychiatric, and motor abnormalities, similar to idiopathic Parkinson’s disease (PD) [4,5,6,7,8]. Chronic exposure of Mn causes toxic Mn accumulation in brain regions, especially in striatum [9,10,11]. Mn toxicity has been identified through overexposure of Mn in occupational (e.g., welders and smelters), environmental, medical and dietary routes [6,8,12]. It has been noted that Mn causes toxic effect mainly in the CNS and lungs, as well as in heart, liver, and reproductive organs and during embryonic stage [4,7,13,14,15,16,17,18,19,20,21]. Some countries use anti-knock agent methylcyclopentadienyl manganese tricarbonyl as a fuel additive, which could increase Mn overexposure to human [22,23]. A number of studies have identified the possible underlying mechanisms of Mn-induced neurotoxicity with some different aspects but it remains unclear. Mn has the ability to induce reactive oxygen species (ROS) generation, lead to mitochondrial dysfunction, impairs endoplasmic reticulum (ER) homeostasis and promotes apoptosis [24,25,26,27]. Mn can also induce excitotoxic cell death through alteration of neurotransmitters levels [28,29,30]. Mn can induce protease activation and apoptotic cell death [31,32]. Recently, involvement of ER stress and ER stress-mediated apoptosis has been found in Mn-induced neurotoxicity in the rat striatum in vivo [33].

The Korean prostrate spurge *Euphorbia supina* (*E. supina)*, in the family Euphorbiaceae, is characterized as a broadleaf weed, with pinkish stems, dense hair and spotted spurge exude a milky sap when injured. It has been used as folk medicine against various diseases such as bronchitis, hepatitis, hemorrhage, etc. It was reported that the plant contained a variety of biologically active components, such as terpenoids, tannins, and polyphenols [34]. Polyphenols have a great interest to researcher as they possess many biological benefits to human health, especially in neurodegenerative diseases including PD and Alzheimer’s disease (AD) [34,35,36]. *E. supine* is abundant in polyphenols and, by using high-performance liquid chromatography-tandem mass spectrometry (HPLC-MS/MS), nine biologically interesting polyphenols were isolated and identified from this plant: gallic acid, protocatechuic acid, nodakenin, quercetin 3-*O*-hexoside, quercetin 3-*O*-pentoside, kaempferol 3-*O*-hexoside, kaempferol 3-*O*-pentoside, quercetin and kaempferol [37]. Polyphenols such as quercetin and kaempferol derivatives from *E. supina* have strong antioxidant properties [37]. Recently, it has been found that polyphenolic compounds of *E. supina* markedly inhibit metastatic cancer in MDA-MB-231 breast cancer cells [38]. A number of studies identified strong antioxidant activity of *E. supina* in presence of several key polyphenols [37]. Moreover, no systematic studies have been conducted to validate the pharmacological efficacy of polyphenols of *E. supina*. The present study aimed to investigate the protective effect of polyphenols of *E. supina* on Mn-induced oxidative stress and the underlying mechanism in human neuroblastoma SKNMC cells and Sprague-Dawley (SD) male rats.

## 2. Results

### 2.1. Total Phenol and Flavonoid Content

The PPEES possessed significant amount of total phenol and flavonoid content expressed as gallic acid and quercetin equivalents, respectively (Table 1). The phenolic content of PPEES was 175.53 ± 5.94 mg GAE/g. Flavonoid content of the PPEES was 98.48 ± 17.73 mg QE/g.

### 2.2. DPPH Scavenging and RPC of PPEES

The DPPH activity of PPEES was found to increase in dose dependent manner. The IC_50_ value of the PPEES was 145.04 ± 6.2 μg/mL, while the IC_50_ of ascorbic acid was 14.27 ± 1.06 μg/mL. The RPC of PPEES was found to increase in dose dependent manner. The IC_50_ value of the PPEES was 86.052 ± 3.94 μg/mL, while the IC_50_ of ascorbic acid was 10.05 ± 0.64 μg/mL In comparison to ascorbic acid, PPEES showed strong antioxidant activity, as determined using DPPH and RPC (Table 2).

### 2.3. Effect of PPEES on SKNMC Cell Lines

The cytotoxic effect of the PPEES on human neuroblastoma cell line SKNMC was evaluated by incubating it with various concentrations of extract (1–1000 µg/mL). The toxicity results revealed a decrease in percentage of viability at higher concentrations of the extract and the IC_50_ value was found to be 1181.281 ± 8.1 µg/mL.

### 2.4. Protective Effect of PPEES on Mn-Induced Cytotoxicity

**Figure 1:**
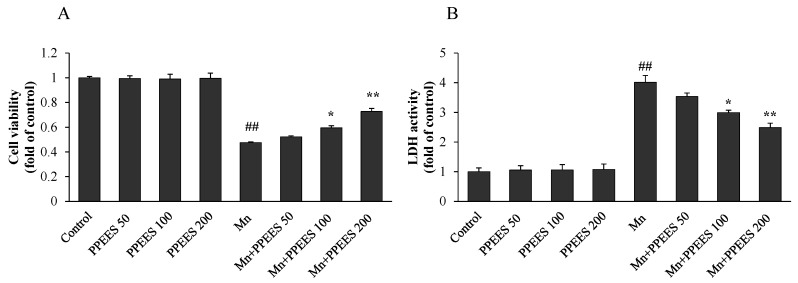
Protective effect of PPEES on Mn -induced cytotoxicity in SKNMC cell lines: (**A**) cell viability; and (**B**) LDH activity. Values were represented as mean ± SD (*n* = 3). ## *p* < 0.01 as compared with the control group; * *p* < 0.05; ** *p* < 0.01 as compared with the Mn alone group.

The effect of PPEES on the viability of SKNMC cells under Mn-induced toxicity conditions was measured by crystal violet assay. Pretreatment of SKNMC cells with PPEES at concentrations of 50–200 µg/mL significantly (*p* < 0.05 or *p* < 0.01) protected SKNMC cells from Mn toxicity. An increase in cell viability was observed in treated cells compared to Mn alone group (Figure 1A). The result displayed that PPEES doses of 100 μg/mL and 200 μg/mL possessed the best protective effects. Correspondingly, PPEES pretreatment significantly decreased (*p* < 0.05 or *p* < 0.01) the Mn-caused LDH release (Figure 1B). No change of the cell viability and LDH activity was observed in control and PPEES groups (Figure 1).

### 2.5. PPEES Attenuated Mn-Induced Oxidative Stress in SKNMC Cells

**Figure 2:**
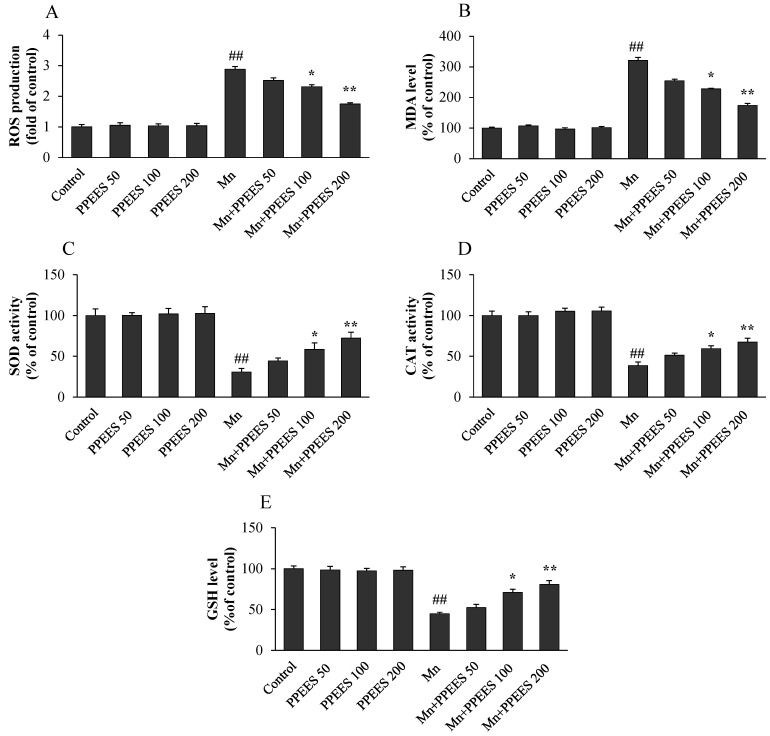
Protective Effect of PPEES on Mn-induced oxidative stress in SKNMC cell lines: (**A**) ROS; (**B**) malondialdehyde (MDA) levels; (**C**) superoxide dismutase (SOD) activity; (**D**) catalase (CAT) activity; and (**E**) glutathione (GSH) levels. Values were represented as mean ± SD (*n* = 3). ## *p* < 0.01 as compared with the control group; * *p* < 0.05 and ** *p* < 0.01 as compared with the Mn alone group.

As shown in Figure 2A, the intracellular ROS level was markedly increased to 2.88-fold (*p* < 0.01) in SKNMC cells with the treatment Mn compared to the control. PPEES pretreatment with different concentrations (50, 100 and 200 μg/mL) significantly reduced the ROS level to 2.51, 2.31 (*p* < 0.05), and 1.75 fold (*p* < 0.01) of the control value, respectively. Similarly, the cells were pretreated with different concentrations of PPEES (50, 100 and 200 μg/mL) in the presence of Mn (500 μM) for 24 h significantly reduced (*p* < 0.01) the MDA levels from 309.08% to 254.81%, 227.71% (*p* < 0.05) and 174.15% (*p* < 0.01) (Figure 2B), respectively. Correspondingly, pretreatment of PPEES at the concentration of 100 and 200 μg/mL significantly increased the activities of SOD and CAT and the GSH level (*p* < 0.05 or *p* < 0.01) (Figure 2C–E). PPEES treatment alone at 50, 100 and 200 μg/mL had no effect on cellular oxidative stress.

### 2.6. PPEES Attenuates Mn-Induced Mitochondrial Dysfuction

**Figure 3:**
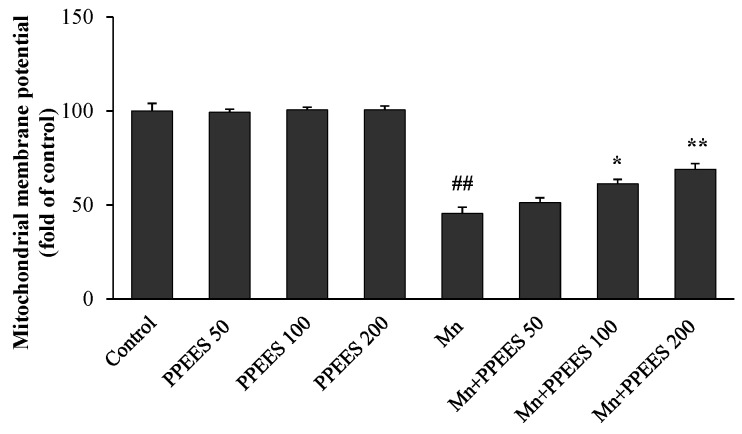
The protective effect of PPEES against Mn-induced mitochondrial dysfunction in SKNMC cells. JC-1 fluorescent dye was used to measure the loss of mitochondrial membrane potential (ΔΨ_m_). Values were represented as mean ± SD (*n* = 3). ## *p* < 0.01, compared to the control group; * *p* < 0.05; ** *p* < 0.01, compared to the Mn alone group.

The loss of mitochondrial membrane potential (ΔΨ_m_) was observed using JC-1, a sensitive fluorescent dye. Mn exposure significantly reduced (*p* < 0.01) the ΔΨ_m_ value in SKNMC cells (Figure 3). In comparison with the control group, the Mn group showed a reduced ΔΨ_m_ at 45.5%, which could be rescued to 51.36%, 61.34% (*p* < 0.05) and 70.94% (*p* < 0.01) with the pretreatment of PPEES at the concentrations of 50, 100 and 200 μg/mL, respectively. No change of ΔΨ_m_ was observed in control and PPEES alone groups (Figure 3). 

### 2.7. PPEES Reduced Apoptosis on Manganese-Induced Apoptosis in SKNMC Cells

**Figure 4:**
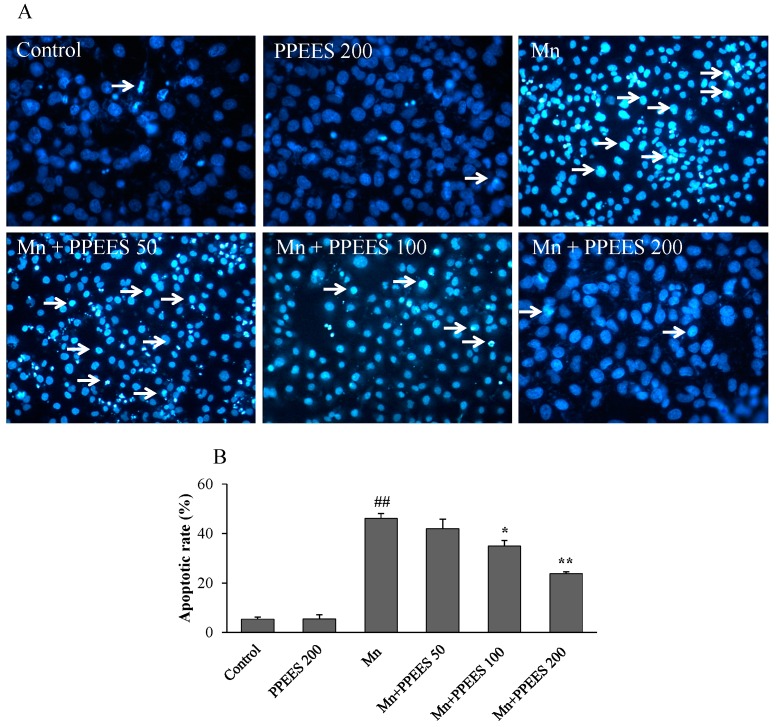
Protective effects of PPEES against Mn-induced apoptosis in SKNMC cells: (**A**) representative pictures showing the apoptotic cells (Hoechst-positive cells) in arrowheads; and (**B**) representative percentage of the apoptotic rate, measured by calculating the percent of Hoechst positive cells over the total number of cells. Values were represented as mean ± SD (*n* = 3). ## *p* < 0.01, compared to the control group; * *p* < 0.05; ** *p* < 0.01, compared to the Mn alone group.

SKNMC cells treated with Mn (500 μM) for 24 h showed typical properties of apoptosis, including chromatin condensation, fragmentation and nuclei shrinkage using Hoechst 33342 staining (Figure 4A). The amount and rate of apoptotic cells were significantly increased (*p* < 0.01) compared to the control and PPEES alone groups. However, the number of apoptotic cell was significantly reduced (*p* < 0.05 or *p* < 0.01) with PPEES pretreatment at 100 and 200 μg/mL in the presence of Mn (Figure 4B). 

### 2.8. PPEES Decreased Mn-Induced ER Stress and ER Stress-Mediated Apoptosis

**Figure 5:**
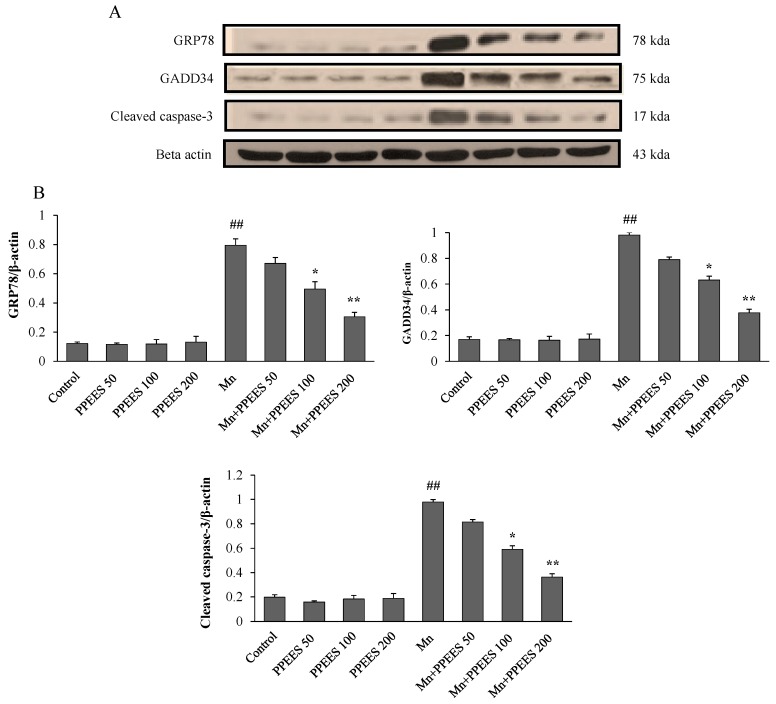
(**A**,**B**) Western blotting was performed to examine the effect of PPEES on the increased protein expression of GRP78, GADD34 and cleaved caspase-3, induced by administration of MnCl_2_ (500 µM). Protein expression was normalized against β-actin. Values were represented as mean ± SD (*n* = 3). ## *p* < 0.01, compared to the control group; * *p* < 0.05; ** *p* < 0.01, compared to the Mn group.

**Figure 6:**
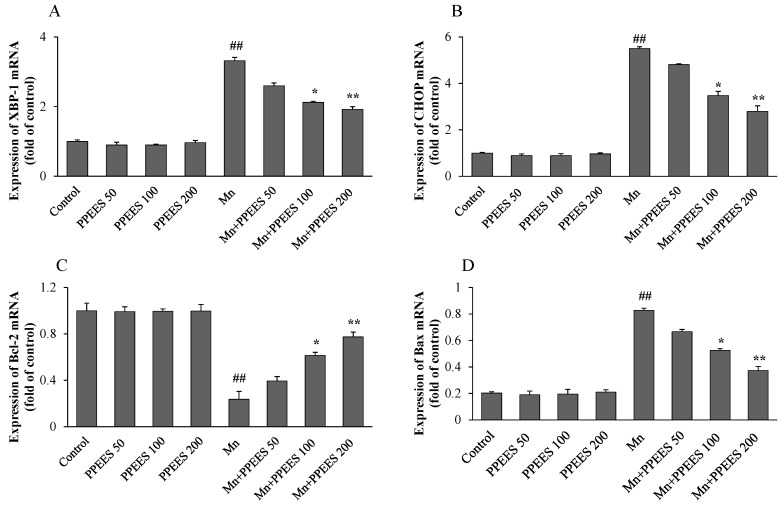
(**A**–**D**) RT-PCR was performed to examine the effect of PPEES on the mRNA expression of XBP-1, CHOP, Bcl-2 and Bax in SKNMC cell that result from Mn treatment. GAPDH served as an internal control. The transcriptive levels of XBP1-1, CHOP, Bcl-2 and Bax were normalized against GAPDH. Values were represented as mean ± SD (*n* = 3). ## *p* < 0.01, compared to the control group; * *p* < 0.05; ** *p* < 0.01, compared to the Mn group.

Western blot and RT-PCR analyses were performed to investigate the effects of PPEES on Mn-induced ER stress and ER stress-mediated apoptosis. Western blot analyses were performed to investigate the effects of Mn and PPEES on the expression of GRP78, GADD34 and cleaved caspase-3 proteins in the SKNMC cell line. The ER stress biomarkers GRP78 and GADD34 were markedly increased (*p* < 0.01) in Mn-treated group, while PPEES pretreatment significantly reduced (*p* < 0.05 or *p* < 0.01) the Mn-induced changes in GRP78 and GADD34 to levels similar to that of both untreated control and PPEES treated only groups (Figure 5). The results showed that the Mn administration significantly increased (*p* < 0.01) the levels of apoptotic hallmark protein cleaved caspase-3 and that the treatment with PPEES significantly reduced (*p* < 0.05 or *p* < 0.01) the cleaved caspase-3 to levels similar to that of both untreated control and PPEES-treated only groups (Figure 5). RT-PCR analyses were performed to investigate the effects of Mn and PPEES on the expression of mRNA levels of XBP-1, CHOP, Bcl-2 and Bax in the SKNMC cell line. Our results showed that compared with the control treatment, the Mn treatment significantly increased (*p* < 0.01) the mRNA expression of XBP-1, CHOP and Bax, while significantly decreased (*p* < 0.01) the mRNA expression of the anti-apoptotic protein Bcl-2 (Figure 6). Interestingly, the treatment with PPEES significantly reversed (*p* < 0.05 or *p* < 0.01) the Mn-induced changes in the XBP-1, CHOP Bax and Bcl-2 mRNA expression levels (Figure 6).

### 2.9. The OF Test

**Figure 7:**
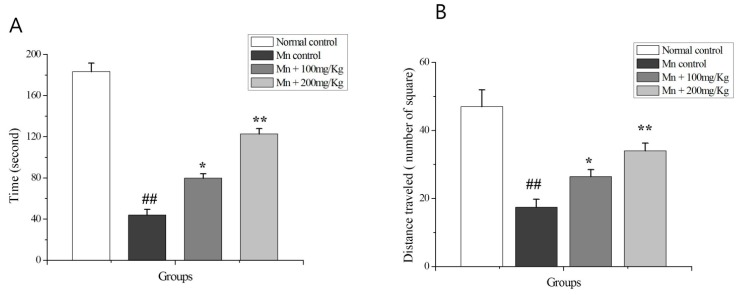
The locomotors activity of normal control, Mn control and PPEES-treated rats was evaluated by open field OF test: (**A**) representation of time spent in the center of the arena; and (**B**) representation of number of squares traveled. Values were represented as mean ± SD (*n* = 5). ## *p* < 0.01 (normal control versus Mn-exposed rats), * *p* < 0.05, ** *p* < 0.01 (Mn-exposed versus Mn + PPEES rats).

The OF test showed that Mn-treated rats spent significantly less (*p* < 0.01) time in the center of arena (Figure 7A) and displayed significantly less (*p* < 0.01) locomotors activity (Figure 7B) compared to normal control and Mn + PPEES groups. PPEES treatment significantly improved (*p* < 0.05 or *p* < 0.01) the time spent in the center area (*p* < 0.05 or *p* < 0.01) and the locomotors activity compared to the Mn-treated animals. To avoid possible unwanted olfactory influences on tested animals, the OF was thoroughly cleaned with a 10% ethanol solution.

### 2.10. The Beneficial Effect of PPEES Treatment on Mn-Induced Histopathological and Immunohistochemically Altered Rats Brain

**Figure 8:**
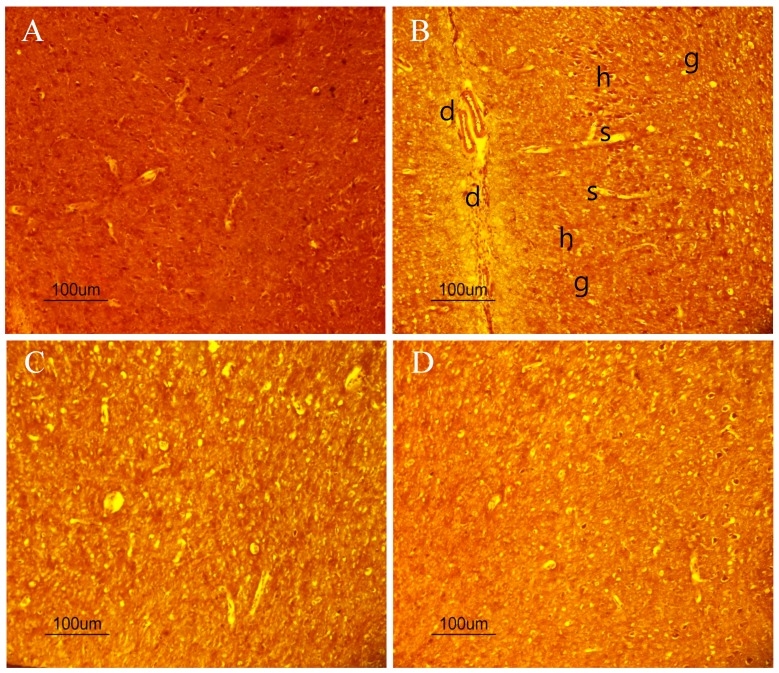
Histopathological images showing the beneficial effects of PPEES on Mn-induced changes in rat striatum: (**A**) control group (Normal saline); (**B**) manganese chloride (15 mg/kg) treated group; (**C**) manganese chloride (15 mg/kg) + PPEES (100 mg/kg); and (**D**) manganese chloride (15 mg/kg) + PPEES (200 mg/kg) treated group (magnification at 10×). Damage (d); Ghost cells (g); hemorrhage (h); and vacuolated cytoplasm (s) (magnification at 10×).

The histopathology examination was conducted using striatum part of brain under microscope (Figure 8A–D). Exposure of Mn led to marked histopathological alterations in the striatum characterized by neuronal damaged and present of ghost cells, hemorrhage and vacuolated cytoplasm. PPEES treatment showed beneficial effect compared to Mn-treated group. There was no histopathological alteration in striatum of normal control (Figure 8A).

**Figure 9:**
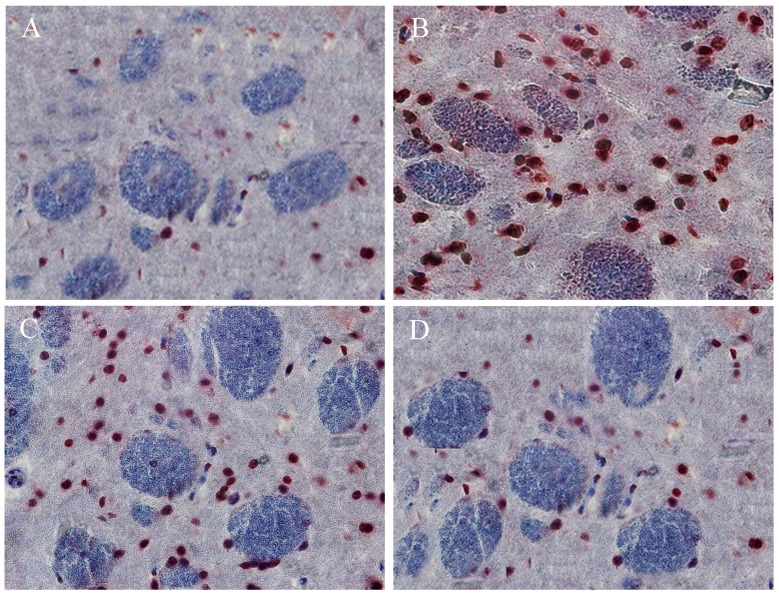
IHC staining showing the protective effect of PPEES against Mn-induced neurodegenetive disease by reducing oxidized RNA in neurons. PPEES treatment significantly reduced 8-hydroxy-2′-deoxyguanosine (8-OHdG) expression that result from Mn exposure in striatum: (**A**) control group (Normal saline); (**B**) manganese chloride (15 mg/kg) treated group; (**C**) manganese chloride (15 mg/kg) + PPEES (100 mg/kg); and (**D**) manganese chloride (15 mg/kg) + PPEES (200 mg/kg) treated group (magnification at 40×).

**Figure 10:**
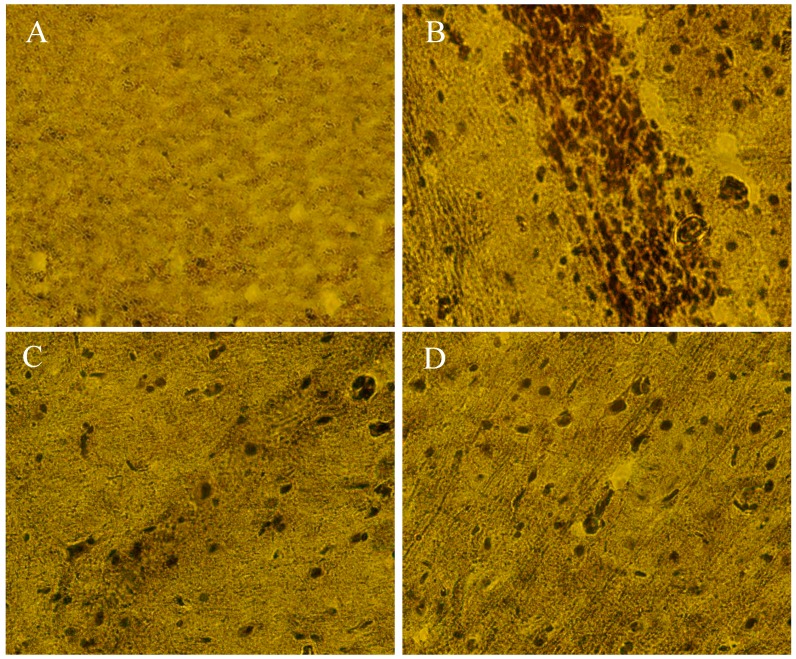
Photographs showing protective effect of PPEES against Mn-induced neurotoxicity by reducing Bax expression in cortex of rats: (**A**) control group (Normal saline); (**B**) manganese chloride (15 mg/kg) treated group; (**C**) manganese chloride (15 mg/kg) + PPEES (100 mg/kg); and (**D**) manganese chloride (15 mg/kg) + PPEES (200 mg/kg) treated group (magnification at 40×).

8-OHdG and Bax immunoreactivity was significantly increased in Mn-treated rats brain with respect to control group (Figure 9B and Figure 10B). Moreover, PPEES treatment significantly reduced Mn-induced immunoreactivity in rat brain (Figure 9C,D and Figure 10C,D). In normal control rats, there was no immunoreactivity (Figure 9D and Figure 10D).

## 3. Discussion

To investigate the protective role of PPEES on Mn-induced neurotoxicity, we used human neuroblastoma SKNMC cells and SD male rats. Our results revealed that Mn treatment could induce ROS generation, oxidative stress, mitochondrial dysfunction, apoptosis and neurotoxicity, while PPEES treatment could effectively resolved these undesired neurotoxicity.

We determined the beneficial effects of PPEES against the Mn-induced toxicity in SKNMC cells. The SKNMC cells were treated with Mn (500 µM) and three different concentrations (50, 100 and 200 µg/mL) of PPEES for 24 h. The results showed that PPEES at all three concentrations was nontoxic to SKNMC cells and that there was no significant difference between the cell viability and LDH activity of the control cells and the PPEES alone cells (Figure 1). Mn exposure at 500 µM significantly decreased (*p* < 0.01) cell viability and increased (*p* < 0.01) LDH activity in SKNMC cells after 24 h. However, pretreatment with PPEES at 100 mg/mL and 200 mg/mL concentrations significantly increased (*p* < 0.05 or *p* < 0.01) the cell viability and decreased (*p* < 0.05 or *p* < 0.01) LDH activity of Mn-exposed cells, which could protect the SKNMC cell from cytotoxicity [39].

To understand the antioxidant effects of PPEES against the Mn-induced oxidative stress, we quantified the intracellular ROS, MDA and GSH level and SOD and CAT activities in vitro. Mn treatment resulted in a significant increase (*p* < 0.01) in the ROS and MDA levels, decreased (*p* < 0.01) GSH levels, and decreased SOD and CAT activities compared with the control treatment and PPEES alone treatment. All three different concentrations (50, 100 and 200 µg/mL) was nontoxic to SKNMC cells and the concentrations of PPEES (100 mg/mL and 200 mg/mL) significantly reduced (*p* < 0.05 or *p* < 0.01) the ROS and MDA levels, increased (*p* < 0.05 or *p* < 0.01) GHS levels, and increased SOD and CAT activities, which suggested PPEES acted as a potent antioxidant (Figure 2) [39,40].

The main target of ROS-induced oxidative damage is mitochondria [41,42]. In the present study, Mn treatment significantly decreased (*p* < 0.01) ΔΨ_m_ of SKNMC cells (Figure 3), which led to mitochondrial dysfunction. PPEES pretreatment significantly attenuated (*p* < 0.05 or *p* < 0.01) the disruption of ΔΨ_m_, which led to initiating mitochondria mediated apoptosis through intrinsic and extrinsic apoptotic [43].

There are three main apoptosis pathways: mitochondrial pathway, death receptor pathway, and ER pathway [44]. It has been demonstrated that Mn treatment could induce apoptosis in SKNMC cell via the involvement of ER stress and mitochondria dysfunction [24]. In the present study, we found that Mn treatment significantly increased (*p* < 0.01) the apoptotic rates [44]. Interestingly, pretreatment of PPEES attenuated (*p* < 0.05 or *p* < 0.01) Mn-induced cytotoxicity involving the inhibition of cell apoptosis (Figure 4).

To investigate the protective role of PPEES on Mn-induced ER stress and ER stress-mediated apoptosis markers (GRP78, GADD34, XBP-1, CHOP, Bcl-2, Bax and cleaved caspase-3), both western blot and RT-PCR analyses were performed. ER stress-signaling pathways regulated by GRP78, leading to UPR survival, cell fate and apoptosis responses [45]. ER stress induces XBP-1 to produce a more active transcription factor [46]. Induction of CHOP expression is most sensitive to ER stress condition and led to DNA damage [45]. Overexpression of GADD34 can initiate or enhance apoptosis through various signals [47,48]. It has been reported that the pro-apoptotic Bax and the anti-apoptotic Bcl-2 proteins regulate the mitochondrial apoptotic pathway [49,50]. Both intrinsic and extrinsic apoptotic pathways can activate caspase-3, a key biomarker of apoptosis and consequently lead to DNA breakdown [43,51]. Our results showed that the Mn administration significantly (*p* < 0.01) altered the GRP78, XBP-1, CHOP, Bcl-2, Bax and caspase-3 activities. Importantly PPEES pretreatment significantly reversed (*p* < 0.05 or *p* < 0.01) the Mn-induced alteration in the GRP78, GADD34, XBP-1, CHOP, Bcl-2, Bax and caspase-3 activities (Figure 5 and Figure 6).

To further understand this, we investigated the protective effects of PPEES on Mn-induced neuronal toxicity in the striatum and cerebral cortex of SD male rats treated with Mn at a dose regimen of 15 mg/kg. The striatum and cerebral cortex parts were chosen because Mn affects more severely the striatum and cortex regions than any other region of the CNS [33]. Exposure of Mn caused histopathological alterations in the striatum. Administration of PPEES (100 and 200 mg/kg) to Mn-exposed rats showed improvement of histopathological alteration in comparison to Mn-treated rats (Figure 8).

Oxidative stress is one of the most important factors in the pathogenesis of neurological disorders (e.g., AD and PD), and 8-OHdG is a common oxidative stress marker produced by oxidation of DNA bases [52]. We found that 8-OHdG expression in striatum region was significantly increased in Mn-exposed rats compared to normal control rats. 8-OHdG expression was significantly reduced in PPEES-treated rats (Figure 9). It has been established that several observed alterations of neuron in the vulnerable brain regions of AD patients are due to upregulation of Bax immunoreactivity [53,54]. Administration of PPEES to Mn-exposed rats showed significant reduction of Bax immunoreactivity (Figure 10).

## 4. Materials and Methods

### 4.1. Plant Material

*E. supina* plants were purchased from the Jirisan Medicinal Herbs (Jirisan, Republic of Korea). The identities of the plants were authenticated by Professor Chang Young-Nam, Ph.D., Department of Chinese Medicine Resource, College of Environmental & Bioresource sciences, Chonbuk National University, Jeonju, Republic of Korea. A voucher of herbarium sheet was deposited in the above-mentioned entity. The aerial parts of *E. supina* were separated and air dried without exposure to sunlight at room temperature for one week to make coarse powder. The coarse powder (500 g) was macerated with 2000 mL of hydro-alcoholic mixture (water:methanol 30%:70% vol/vol) for 10 days at room temperature. The resulted solution was filtered through Whatman filter paper (No. 4) and concentrated using a rotary evaporator under reduced pressure at <35 °C. The total dried plant extract was storage for further stage.

### 4.2. Extraction of Polyphenol Enriched Extracts of E. supina (PPEES)

To obtain polyphenol extracts of *E. supina* (PPEES), the total plant extract residue was first dissolved in water and 200 mL of petroleum ether (four times) was added to obtain a clear upper layer (petroleum ether). Then lower layer (aqueous water layer) was washed with 200 mL of ethyl acetate containing glacial acetic acid (10 mL/L) (four times). Finally, the resulted solutions were combined, and ethyl acetate was evaporated to obtain PPEES, which was stored at −20 °C [55]. Details of the Identification of polyphenolic compounds are described in the Supplementary Materials (Table S1, Figure S1).

### 4.3. Total Phenolic Content (TPC)

The total phenolic content of the PPEES was measured using the Folin–Ciocalteu reagent [56,57]. Briefly, PPEES was oxidized by adding Folin–Ciocalteu reagent and then sodium carbonate was added to neutralize the reaction. After 30 min, the absorbance of resulting solution was read at 760 nm using gallic acid as standard. Total phenolic content was expressed as mg gallic acid equivalent (GAE)/gm of extract.

### 4.4. Total Flavonoid Content (TFC)

The flavonoid content was determined using quercetin (Q) as reference standard [58,59]. The PPEES was mixed with aluminum trichloride and one drop of acetic acid was added. Then, the mixture was diluted with ethanol. After 40 min, the absorbance of resulting solution was measured at 415 nm. The absorbances of blank sample and standard quercetin solution were measured under the same condition. Total flavonoid content was expressed as mg quercetin equivalent (QE)/gm of extract.

### 4.5. 1,1-Diphenyl-2-picrylhydrazyl (DPPH) Assay

The 1,1-diphenyl-2-picrylhydrazyl (DPPH) radical scavenging activity assay was conducted following previously described methods with minor adjustments [60]. The PPEES was mixed with the 0.004% methanol solution of DPPH and incubated for 30 min. After incubation, the absorbances of all the samples were determined at 517 nm. Inhibition of DPPH (D) was measured using Equation (1).

D(%) = {(Ac − As)/Ac} * 100
(1)

where Ac and As are the absorbance of control and test samples, respectively.

### 4.6. Reducing Power Capacity (RPC)

The assay was conducted following previously described method based on the measurement of absorbance of Pearl’s Prussian blue [61]. The PPEES was added to a mixture of PBS (pH 6.6) and potassium ferricyanide [K_3_Fe(CN)_6_]. The resultant mixture was incubated and centrifuged after adding trichloroacetic. The upper layer was mixed with distilled water and FeCl_3_. The absorbance was measured at 700 nm.

### 4.7. Cell Culture

SKNMC, a human neuroblastoma cell line, was obtained from the American type culture collection (Manassas, VA, USA). The cells were grown in Dulbecco’s modified eagle medium (DMEM) supplemented with 10% fetal bovine serum (FBS), 4.5 g/L d-glucose, 2 mmol/L l-glutamine, 110 mg/L sodium pyruvate, 100 U/mL penicillin, and 100 μg/mL streptomycin at 37 °C in a humidified atmosphere containing 95% air and 5% CO_2_.

### 4.8. Cytotoxicity of PPEES

PPEES was dissolved in dimethyl sulphoxide (DMSO) (D2650, Sigma, Saint Louis, MO, USA) to obtain a stock solution of 20 mg/mL, and 0.2 mg/mL of sub stock solution was prepared by diluting 10 μL of the stock solution into 990 μL serum-free DMEM medium, and prepared at different concentrations (the percentage of DMSO in the experiment should not exceed 0.5). Stock and sub stock solutions were both stored at 4 °C. Cell viability was determined by crystal violet assay. Briefly, SKNMC cells were seeded onto 24-well plate (5 × 10^4^ cells/well), incubated overnight and pretreated with various concentrations of PPEES (0–1000 μg/mL) for 24 h. Then, the medium was removed and cells were washed with phosphate buffer solution (PBS). Two hundred microliters of 0.2% crystal violet solution was added to each well and incubated for 10 min at room temperature then washed with water and 100 μL 1% SDS was added to solubilize the stain solution until color was uniform and there were no areas of dense coloration in bottom of wells. The samples were read at 590 nm in microplate reader (Spectra MAX, Gemini EM, Molecular Device, Sunnyvale, CA, USA).

### 4.9. Lactate Dehydrogenase (LDH) Activity

The LDH activity assay was conducted based on reduction of nicotinamide adenine dinucleotide (NAD) by LDH. LDH release into the media was taken as an indicator of cell damage and the assay is based on the principle of reduction of nicotinamide adenine dinucleotide (NAD) by LDH. The stoichiometric conversion of a tetrazolium dye utilized the reduced NAD (NADH) that was measured spectrophotometrically using an assay kit Tox-7 (Sigma, Saint Louis, MO, USA). Briefly, SKNMC cells were seeded (5 × 10^4^ cells/well)) and cultured in 24-well culture plates. The cells were then preincubated with or without different concentrations of PPEES (50, 100, and 200 μg/mL) at 37 °C for 6 h followed by incubation with 500 μM MnCl_2_ (CAS: 7773-01-5, Sigma, Saint Louis, MO, USA) for 24 h. After treatment, cells were centrifuged at 240× *g* for 4 min and supernatant solution was transferred to assay plate. The plate was wrapped in foil and incubated at room temperature for 30 min. After incubation, by adding stop solution the reaction was terminated and the plate read at 490 nm and at a reference wavelength of 690 nm in microplate reader (Spectra MAX, Gemini EM, Molecular Device, Sunnyvale, CA, USA). The amount of LDH release is expressed as the fold of absorbance of control.

### 4.10. Cell Viability

Mn-induced cell survival was determined by crystal violet assay. Briefly, SKNMC cells were seeded (5 × 10^4^ cells/well)) and cultured in 24-well culture plates. The cells were then preincubated with or without different concentrations of extract (50, 100, and 200 μg/mL) at 37 °C in a humidified atmosphere of 5% CO_2_/95% air for 6 h followed by incubation with 500 μM Mn for 24 h. Afterwards, the medium was removed and cells were washed with phosphate buffer solution (PBS) and 200 μL of 0.2% crystal violet solution was added to each well and incubated for 10 min at room temperature, and then wash with water and 100 μL 1% SDS was added to solubilize the stain solution until the color was uniform and there were no areas of dense coloration in bottom of wells. The samples were read at 590 nm in a microplate reader (Spectra MAX, Gemini EM, Molecular Device, Sunnyvale, CA, USA).

### 4.11. Measurement of Intracellular Reactive Oxygen Species (ROS) Level

The intracellular ROS generation was measured based on enzymatic conversion of a non-fluorescent compound dichloro-dihydro-fluorescein diacetate (DCFH-DA) to highly fluorescent compound DCF, following the previously described method [62]. Briefly, the cells were harvested and seeded onto 6-well plate with 2 × 10^5^ cells per well in culture media and allowed to attach overnight. The cells were pretreated with the doses of PPEES at 50, 100 and 200 µg/mL at 37 °C for 6 h and washed with PBS. Then, the cells were treated with Mn (500 µM) for additional 24 h. Finally, after washing, the cells were seeded on the 6-well plate with PBS once and incubated with DCFH-DA (10 μmol/L) for 30 min at 37 °C in the dark. The fluorescence intensity was measured in the microplate reader (Spectra MAX, Gemini EM, Molecular Device, Sunnyvale, CA, USA) at an excitation wave length of 485 nm and an emission wave length of 538 nm after the cells were washed three times with PBS to remove the extracellular DCFH-DA. The level of intracellular ROS is shown as a fold of control.

### 4.12. Antioxidant Status

Antioxidant status of PPEES was examined by measurement of intracellular malondialdehyde (MDA) and glutathione (GSH) levels, and superoxide dismutase (SOD) and catalase (CAT) activities, using specific assay kits (Nanjing Jiancheng Co., Ltd., Nanjing, China) according to the manufacturer’s instructions. In brief, SKNMC cells were seeded (2 × 10^5^ cells/well) into 12-well plates and pre-treated with PPEES (50, 100 or 200 µg/mL) at 37 °C for 6 h. The cells were incubated with or without Mn (500 µM) for 24 h after removing PPEES containing medium. Then cells were washed with cold PBS and lysed using the cell lysis buffer. The cell lysates were centrifuged at 14,000× *g* for 10 min at 4 °C and supernatant solutions were used for measuring the levels of MDA and GSH, and the activities of SOD and CAT. Protein concentrations were measured using the BCA protein assay kit (Intron Biotechnology, Inc., Gyeonggi, Korea).

### 4.13. Measurement of Mitochondrial Membrane Potential (ΔΨ_m_)

Harvested SKNMC cells the day before the experiment and seeded onto 6-well plate with 2 × 10^5^ cells per well in culture media and allowed to attach overnight. The cells were pretreated with the doses of PPEES at 50, 100 and 200 µg/mL at 37 °C for 6 h and washed with PBS. Then, the cells were treated with Mn (500 µM) for an additional 24 h. Finally, after washing, the cells were seeded on a 6-well plate with PBS once and incubated with JC-1 (10 mM final concentration) for 30 min at 37 °C in the dark. The JC-1 green fluorescence intensity was measured in the microplate reader (Spectra MAX, Gemini EM, Molecular Device) at an excitation wave length of 488 nm and an emission wave length of 530 nm after the cells were washed two times with PBS to remove the extracellular JC-1. Monomeric JC-1 green fluorescence emission and aggregate were measured at excitation wavelength 488 nm, emission wavelength 530 nm on a microplate reader (Spectra MAX, Gemini EM, Molecular Device, Sunnyvale, CA, USA).

### 4.14. Apoptosis Assay

Hoechst33342 staining was conducted based on qualitative and quantitative measurements of the apoptotic cells by distinguishing apoptotic cells from normal cells. SKNMC cells were cultured in 6-well plates for 24 h. After treatment, the cells were incubated with 5 μg/mL Hoechst 33342 for 15 min, then washed twice with PBS and finally visualized by inverted fluorescence microscopy (Axioskop 2 plus microscope, Carl Zeiss, Oberkochen, Germany). The apoptotic cells were counted by observation of minimum 200 cells from five non-overlapping fields in all groups, and expressed as a percentage (%) of the total number of cells counted.

### 4.15. Real Time Polymerase Chain Reaction (RT-PCR)

To examine the protective mechanism of PPEES, the expression of X-box binding protein-1 (XBP-1), C/EBP homologous protein (CHOP), Bcl-2 and Bax was measured by real time quantitative polymerase chain reaction (RT-qPCR). The total RNA was extracted from SKNMC cells using trizol reagent (sigma-Aldrich, Saint Louis, MO, USA). The integrity of mRNA was measured spectrophotometrically examined according to its A260/A280 absorption. Subsequently, reverse transcription was used to obtain cDNA. RT-qPCR was conducted on Mastercycler ep realplex (Eppendorf, Hamburg, Germany) using housekeeping gene GAPDH as an internal control. Briefly, the amplification of primer was carried out with 40 cycles at a melting temperature of 94 °C for 15 s, an annealing temperature of 60 °C for 1 min, and an extension temperature of 72 °C for 50 s. The primers used in the amplification were as follow: XBP-1, forward primer: 5′-AAACAGAGTAGCAGCGCAGACTGC-3′, reverse primer: 5′-GGATCTCTAAAACTAGAGGCTTGGTG-3′; CHOP, forward primer: 5′-GAAAGCAGAAACCGGTCCAAT-3′, reverse primer: 5′- GGATGAGATATAGGTGCCCCC-3′; Bcl-2, forward primer: 5′-CCAGGTCTCCGATGAACTTTT-3′, reverse primer: 5′-CAGTGGTTCCATCTCCTTGTTG-3′; Bax, forward primer: 5′-TTTGCTTCAGGGTTTCATCC-3′, reverse primer: 5′-GCCACTCGGAAAAAGACCTC-3′; GAPDH, forward primer: 5′-TGGAGTCTACTGGCGTCTT-3′, reverse primer: 5′-TGTCATATTTCTCGTGGTTCA-3′. The fold or percentage of change in the relative expression of the mRNA of target gene l was measured by the 2^−ΔΔ*C*t^ method.

### 4.16. Western Blotting

The total proteins were extracted from SKNMC cells by using radioimmunoprecipitation assay (RIPA) lysis buffer (Intron Biotechnology, Inc., Gyeonggi, Korea), and the protein concentration was measured using bicinchoninic acid (BCA) kit (Intron Biotechnology, Inc., Gyeonggi, Korea). The separation of proteins was carried out on 8% and 12% polyacrylamide gels, and nitrocellulose (Bio-Rad, Hercules, CA, USA) membranes were used for electro-transferred in a semi-dried environment. Blots were blocked by 5% skim milk (tris-buffer and 0.1% Tween-20) and then incubated with primary anti-GRP78 (1:1000; SC-13539, Santa Cruz Biotechnology, Inc., Dallas, TX, USA), anit-GADD34 (1:1000, ab9869, Abcam, Cambridge, UK) and anti-cleaved caspase (1:1000, Asp175, 9661, cell signaling, Danvers, MA, USA) antibodies at 4 °C overnight. Subsequently, the blots were incubated with anti-mouse (#115-035-003; Jackson ImmunoResearch laboratories, Inc., West Grove, PA, USA), anti-goat (SC-2020, Santa Cruz Biotechnology, Inc.), and anti-rabbit (SC-2004, Santa Cruz Biotechnology, Inc.) secondary antibodies at room temperature for 1 h. Then, the blots were developed with EZ-Western Lumi Plus solution (ATTO Corporation, Tokyo, Japan) (Millipore Corporation, Billerica, MA, USA) and analyzed with Ez-Capture ST (ATTO Corporation, Tokyo, Japan).

### 4.17. Experimental Animal and Treatments

Seven-week-old Sprague-Dawley (SD) male rats, weighing 220–250 g each were purchased from DBL (Eumseong, South Korea). They were kept in clean and dry polypropylene cages with 12-h light–dark cycle at 25 ± 2 °C and 45%–55% relative humidity in the animal house, Pharmacology Department, Chonbuk National University. The rats were fed with a standard laboratory diet and water ad libitum. After a week of adaptation, the rats were randomly divided into four groups (each group, *n* = 5). The protocol used for this study in the rat as an animal model was carried out with the guidelines of the Institutional Animal Care and Usage Committee (IACUC) with approval from ethical committee of Chonbuk National University, Korea for using animals by describing the protocols of the study (Approved number: CBNU 2015-099).

The rats were divided into four groups, each group with 5 rats. Group I for normal control, other groups for Mn which were treated by 15 mg MnCl_2_/kg body weight of rats through intraperitoneal (i.p.) injection five days/week for three weeks. Then, the rats designated for PPEES groups (Group III and IV) followed a daily oral dose of 100 and 200 mg/kg for another four weeks, while the rats in Mn-exposed (group II) and normal control groups received normal saline orally. Details of the treatment pattern and groups are described in the Supplementary Materials. Body weight and food consumption were measured daily (Figures S2 and S3).

### 4.18. The Open Field (OF) Test

The OF test was performed based on observation of locomotor activity of rats in an ideal environment. Briefly, rats were placed in an ideal environment in an OF box for a 30 min session. We recorded the locomotors activity throughout the experiment, spent time and number of squares traveled in the central area of the OF as indices of animal anxiety level.

### 4.19. Collection of Brain

The rats were deeply anesthetized with ketamine and normal saline (0.9%) was used for transcardial perfusion. The brain tissues were fixed using 4% paraformaldehyde (pH 7.4) solution for 12 h; incubated overnight at 4 °C in 100 mM sodium phosphate buffer (pH 7.4) containing 15% sucrose followed by 30% sucrose; and embedded in optimal cutting temperature (OCT, Leica Biosystems Melbourne Pty Ltd., DB Maarn, the Netherland) medium. Coronal sections (20 μm) from cryofixed tissue were collected on silane-coated slides (Muto Pure Chemical Co., Ltd., Tokyo, Japan) and stored at −70 °C.

### 4.20. Histopathology and Immunohistochemistry

Histopathological examination was conducted on the striatum part of brain from SD rats after embedded in OCT medium, following the previously described method [33]. The histopathological alterations were observed using hematoxylin and eosin (HE) staining under light microscope.

To examine the protective effect of the PPEES treatments on Mn-induced immunoreativity of oxidative protein 8-hydroxy-2’-deoxyguanosine (8-OHdG), pro-apoptotic protein Bax and apoptotic protein caspase-3 immunohistochemistry (IHC) was performed in the striatum and cortex of all treatment groups. Sections (14 μm) were prepared from OCT embedded samples described above. The sections were treated with mouse polyclonal anti-8-OHdG (1:500, N45.1, ab48508, Abcam) and rabbit polyclonal anti-Bax (1:500; P-19, sc-526, Santa Cruz Biotechnology, Inc.) antibodies at 4 °C overnight. Subsequently, these were incubated with biotinylated goat anti-mouse (1:30, code: D0314, Dako, Burlington, ON, Canada) and goat anti-rabbit (1:80, code: D0487, Dako) immunoglobulins and latter visualized with substrate chromogen (code: K3464, Dako), followed by hematoxylin and mounted with aqueous mount medium. The sections were cover slipped after drying and observed under a microscope, and images were taken by Nikon Differential Interference Contrast Inverted Microscope (Nikon, Kanagawa, Japan) equipped with Narishige micromanipulators (Narishige, Tokyo, Japan).

### 4.21. Statistical Data Analysis

All data were expressed as mean ± SD and one-way ANOVA (Analysis of variance) followed by Dunnett’s test was used for the statistical analysis using SPSS software (version 16). * *p* < 0.05 and ** *p* < 0.01 were considered significant.

## 5. Conclusions

**Figure 11:**
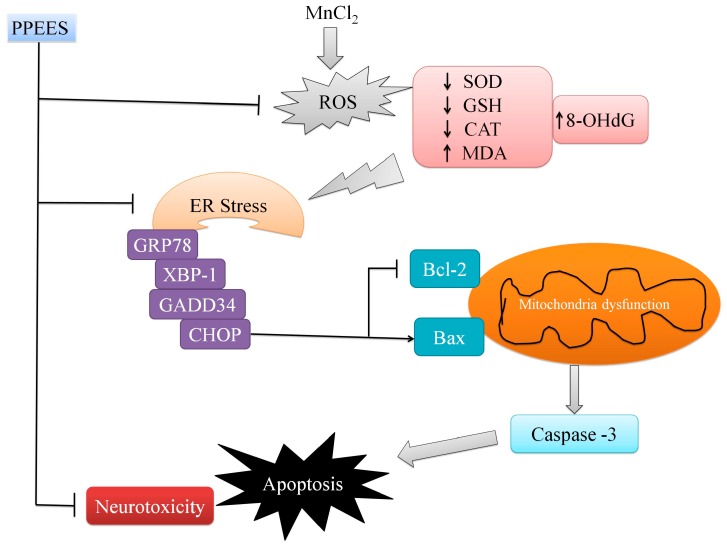
The proposed mechanism of PPEES against Mn-induced toxicity. The schematic diagram shows Mn could exceed ROS; subsequently, altering activity of SOD and CAT. Changing GSH, MDA and 8-OHdG levels led to ER stress, followed by apoptosis through mitochondrial dysfunction. This diagram shows that PPEES prevents the Mn-induced neurotoxicity through regulation of ER stress and ER stress-mediated apoptosis.

In conclusion, the present study reveals that PPEES could effectively inhibit Mn-induced neurotoxicity through antioxidant properties via regulation of ER stress and ER stress-mediated apoptosis (Figure 11). Further studies are also needed to elucidate the precise mechanism of action of PPEES and to evaluate its neuroprotective effects in various neurological disorders.

## Figures and Tables

**Table 1:** Total phenolic content and flavonoid content of PPEES.

TPC in PPEES (mg∙GAE/g)	TFC in PPEES (mg∙QE/g)
175.53 ± 5.94	98.48 ± 7.73

**Table 2:** Antioxidant capacity of PPEES.

DPPH Radical Scavenging Activity; IC50 (µg/mL)		Reducing Capacity of PPEES; IC50 (µg/mL)	
PPEES	Ascorbic acid	PPEES	Ascorbic acid
145.044 ± 6.2	14.27 ± 1.06	86.0517 ± 3.94	10.05 ± 0.64

## Supplementary Materials

The following are available online at www.mdpi.com/1422-0067/18/2/300/s1.
