# Supplementary material for: Polyphenolic Extract of Euphorbia supina Attenuates Manganese-Induced Neurotoxicity by Enhancing Antioxidant Activity through Regulation of ER Stress and ER Stress-Mediated Apoptosis
Source: Int J Mol Sci. 2017 Jan 30;18(2):300. doi: 10.3390/ijms18020300 (PMC5343836; doi:10.3390/ijms18020300)
Supplement: Supplementary file 1 [file ijms-18-00300-s001.pdf]

Supplementary Materials: Polyphenolic Extract of *Euphorbia supina* Attenuates Manganese-Induced Neurotoxicity by Enhancing Antioxidant Activity through Regulation of ER Stress and ER Stress-Mediated Apoptosis

Entaz Bahar, Geum-Hwa Lee, Kashi Raj Bhattarai, Hwa-Young Lee, Min-Kyung Choi, Harun-Or Rashid, Ji-Ye Kim, Han-Jung Chae and Hyonok Yoon

1. Identification of Polyphenolic Compounds by Chromatographic Method

In order to recognize polyphenolic components of PPEES, column chromatography and thin layer chromatography were used. The PPEES was subjected to column chromatography with hexane: ethyl acetate in various solvent ratio. The fractions were collected depending on the visible changes in the colorful bands running out of the column. Then the fractions were subjected for TLC profiling. TLC plate was sprayed by 10% sulphoric acid with heating and observed at 254 and 365 nm wavelength under UV light. The R_f values resulted by test samples were 0.86 and 0.76 for extract which were parallel to the R_f values of reference compounds kaempferol and quercetin (Table S1 & Figure S1).

Table S1. Identification of polyphenols from PPEES.

Compounds	Standards	Results
Polyphenols	Kaempferol (K)	+
	Quercetin (Q)	+

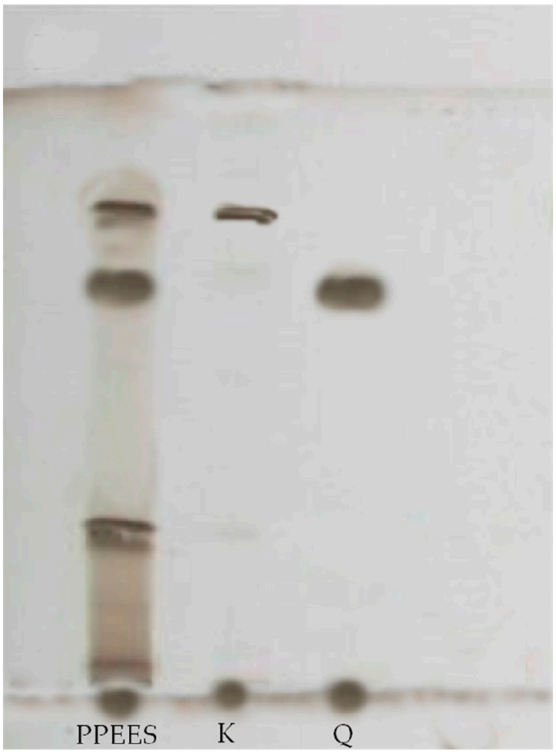

Figure S1. Thin layer chromatography (TLC) of PPEES, Kaempferol (K) and Quercetin (Q).

2. Group Design and Treatment Pattern

The rats were divided into four groups, each group with 5 rats. The groups were designed as follow:

Group I; Normal control: 0.9% normal saline;

Group II; Mn control: 15 mg $MnCl_2$/kg (15 doses);

Group III; Mn + PPEES 100: 15 mg $MnCl_2$/kg (15 doses) + 100 mg/kg (20 doses);

Group IV; Mn + PPEES 200: 15 mg $MnCl_2$/kg (15 doses) + 200 mg/kg (20 doses);

Group I for normal control, other groups for Mn which were treated by 15 mg $MnCl_2$/kg body weight of rats through intraperitoneal (i.p.) injection five days/week for three weeks. Then the rats designed in PPEES groups (Group III and IV) followed by a daily oral dose of 100 and 200 mg/kg. For another four weeks, while the rats in Mn exposed (group II) and normal control groups received normal saline orally at the same time, respectively. The treatment patterns were represented in chart.

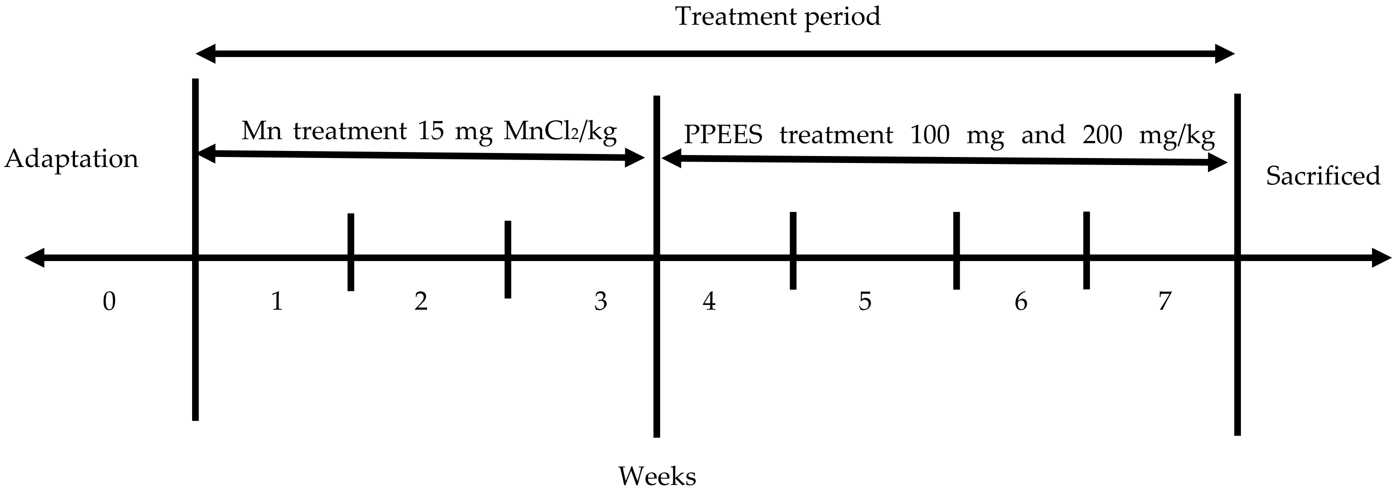

Chart: Experimental treatment of PPEES to the rats.

3. Body Weight and Food Consumption

Rats body weight and food consumption were measured daily. In comparison with normal control group, body weight was slowly increased and food consumption was less during Mn treatment in rats (Figures S2A and S3A).Interestingly, during PPEES treatment rats body weight and food consumption were increased as control group (Figures S2B and S3B).

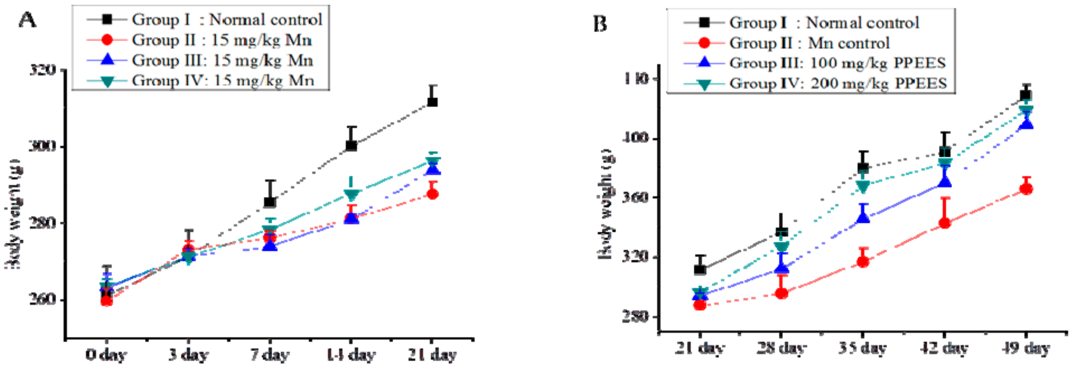

Figure S2. Body weight (g) test in control and Mn-exposed rats. (**A**) Body weight during Mn treatment; and (**B**) Weight during PPEES treatment.

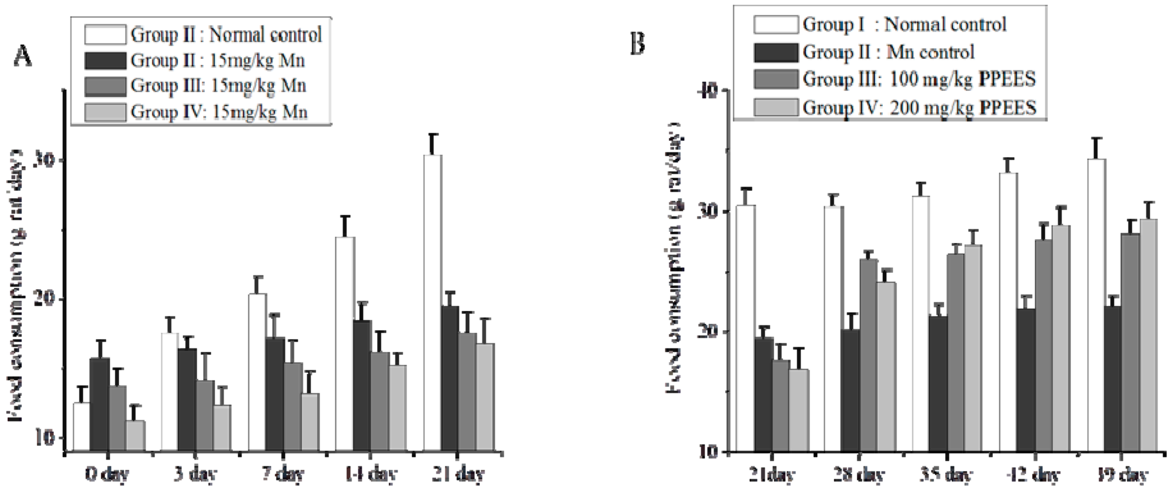

Figure S3. Food consumption (g) test in control and Mn-exposed rats. (**A**) Food consumption during Mn treatment; and (**B**) Food consumption during PPEES treatment.
